# Contemporary Trends and Habits in the Consumption of Sugar and Sweeteners—A Questionnaire Survey among Poles

**DOI:** 10.3390/ijerph16071164

**Published:** 2019-04-01

**Authors:** Marlena Pielak, Ewa Czarniecka-Skubina, Joanna Trafiałek, Artur Głuchowski

**Affiliations:** Department of Food Gastronomy and Food Hygiene, Faculty of Human Nutrition and Consumer Sciences, Warsaw University of Life Sciences, 02-787 Warsaw, Poland; marlenapielak@gmail.com (M.P.); joanna_trafialek@sggw.pl (J.T.); artur_gluchowski@sggw.pl (A.G.)

**Keywords:** sugar, sweeteners, consumers, nutritional habits, Poles

## Abstract

The purpose of the paper was a cross sectional study to evaluate the use of sugars and selected sweeteners by Polish consumers in their diet. The survey was conducted using the direct interview method on the group of 2000 adults declaring the consumption of sugar or sweeteners. The ANOVA test and multi-dimensional cluster analysis was used to the data interpretation (*p* < 0.05). It was stated that the consumption of sugar among consumers remained at a high level. Respondents declared taking up the activities towards reducing sugar intake in their diet mostly due to health-related reasons. It was emphasized in particular by women taking part in the survey. The most frequent way to limit the amount of sugar in the diet consisted in choosing sweeteners, mainly stevia and xylitol. However, the knowledge concerning steviol glycosides among the consumers was not extensive. Results are the source of up-to-date information concerning the consumption of sugar and sweeteners. Consumers to whom nutrition campaigns on the necessity to limit the content of sugar in the diet are worth addressing were identified. A hypothesis, that consumers are currently more aware of the negative influence of increased sugar consumption on their health and they aim at limiting the content of added sugar in their diet, was confirmed.

## 1. Introduction

The diet of Poles, similarly to the majority of inhabitants of EU member states, is characterized by a too-high percentage of energy from refined sugars. Currently, in spite of lower consumption of sugar in its pure form, its total consumption has increased. This results from increased consumption of processed products with the addition of sugar. Taking into account pure sugar and the sugar contained in preserves, in the 1950s its consumption in Poland amounted to 21–27.9 kg/1 inhabitant, and was constantly increasing in order to reach 30.6–38.9 kg in the 1960s, 41.4 kg in the 1980s and 44.4 kg/person/year in 1990. In the last two decades, total sugar consumption oscillated between 38.4–42.3 kg/person/year [1], approximately 115 g per person/day [2]. High annual consumption of added sugar is being recorded all over the world. In the years 2015–2017, it amounted on average to 22.3 kg/person and differed depending on the continent. In Europe it was on average 36.1 kg, in the USA 31.8 kg, in Latin America—41.8 kg, in Asia—18.2 kg (it was higher in Indonesia—25.4 kg), and in Africa—15.7 kg/person/year (the quantity recorded in Egypt—36.7 kg) [3]. Sugar intake in the diet of Poles exceeds the average consumption in other European countries, and is close only to Latin America. The sugar intake in Poland highly exceeds WHO’s recommended level of added sugar intake, which is less than 50 g per day (<10% of total energy intake) [4]. It is important to find out what is the reason for such a significant consumption of sugar, as well as to identify groups of consumers that should be reached with an appropriate campaign promoting healthy behavior. It is also crucial to offer new sweeteners. Therefore, health messages encouraging people to “cut down on sugar” have been lately actively promoted through media and public health interventions [5,6]. 

Within the last 30 years, the trend consisted in replacing slow-release carbohydrate products with highly processed refined sugars. It is connected among others with the increased consumption of saccharose and fructose in fruit juices and beverages as well as confectionery [7]. 

Saccharose as well as products containing this sugar are characterized by fast absorption of glucose in blood. Excessive consumption of sugar (sucrose) and, as a result, overconsumption of energy and energy dense foods reduces the health quality of the diet. It is also a risk factor for several diseases. Sugar overconsumption may lead to a variety of health problems entailing metabolic and psychiatric disturbances in the context of a sedentary lifestyle [8,9]. Among the many diseases are: obesity, cardiovascular disease, type 2 diabetes, diseases of the osteoarticular system, and hormonal disorders [10,11,12,13,14,15]. For this reason, sugar intake above recommended levels is a global public health concern [4]. Increased food awareness and the growing concern consumers of health, as well as the higher incidence of obesity, metabolic syndrome and diabetes has resulted in an increase in interest for foods with reduced sugar [11,14]. Throughout human history, a variety of naturally occurring substances have provided additional sources of sweetness. Among these, for example, licorice root (*Glycyrrihza glabra L*.) has a long history of medicinal use in both Eastern and Western systems of medicine [16].

As a result, the demand for natural sweeteners with low calorific value, acceptable taste and healthy qualities which could replace saccharose is increasing [17]. The consumption of sweeteners has been steadily increasing over the last decades. People have tried to limit sugar intake by using alternative sweeteners: semisynthetic (e.g., lactitol, maltitol, xylitol), synthetic (e.g., aspartame, acesulfame K, saccharinate, cyclamate), as well as natural substances (e.g., thaumatin, kurkulina, steviol glycosides). For determine the precise clinical benefits of reducing added sugars is needed the long-term trials. This is the reason that still looking for novel sweeteners, especially natural sweeteners [18]. Commercial sweeteners present on the market are usually made of a combination of natural and artificial sweet substances. However, it is advisory to eliminate artificial sweeteners from the diet of some people, especially those pregnant and during lactation [19]. Another study confirmed that aspartame has a carcinogenic effect or causes serious health problems [20]. 

Although a number of sweet proteins and other compounds are recognized and have been studied extensively, for the time being relatively few sweet-tasting plant-derived natural products have been launched commercially as sucrose substitutes [21]. Both popular and trade publications include articles about sugars and newer sugar substitutes. The major topic addressed in such articles is the public or personal pressure on the consumer to make him switch from to more natural low- or zero-calorie alternatives. The two botanical sweeteners that have enjoyed a prodigious surge in usage in just a few years are stevia (*S. rebaudiana*) and luo han guo (*S. grosvenorii*) [16].

Steviol glycosides sourced from the *Stevia rebaudiana* plant have recently aroused the interest of technologists. In 2011, they were approved by European Food Safety Authority for use in food [22,23]. As consumer preferences continue to shift toward natural products, the consumption of steviol glycosides is expected to increase than other low-calorie sweeteners [24]. 

Based on the growing number of products with stevia, such as drinks, table-top sweeteners, candy and other processed foods, or various delicacies on the world and Polish market, it is clear that the addition of steviol glycosides can increase the palatability and enjoyment of food by improving flavor and smell [25,26]. In addition, alarming reports about poor eating habits of Poles [27] prompted the study of contemporary trends and nutrition habits in the consumption of sugar and sweeteners.

The purpose of the paper was a cross sectional study to evaluate the use of sugars and selected sweeteners by Polish consumers in their diet. A hypothesis was made that consumers are currently more aware of the negative influence of increased sugar consumption on their health and they aim at limiting the content of added sugar in their diet. 

## 2. Materials and Methods

The survey was conducted using the direct interview method on a group of 2000 adult respondents declaring the consumption of sugar or sweeteners, who agreed to fill in the survey questionnaire consisting of two parts. Analyses related to this paper were designed as a study with a convenience sampling. 

The respondents were free to participate in the research. Because the research was non-invasive and details the participants remained undisclosed, the research does not fall within the remit of the Helsinki Declaration.

### 2.1. Data Collection

To collect data a questionnaire was used. The questionnaire was validated by means of a pilot study in 20 people. All problems have been identified. For example, unintelligible questions or construction of questionnaire. The questionnaire was completed and amended. The data was collected by well-trained researchers who are the authors of this manuscript, during one-to-one appointments. Each respondent who had agreed to take part in the study by providing informed consent, was invited to fulfill the questionnaire. Explanations were given if necessary. 

The participants were a convenient sample of consumers, from the central part of Poland. The inclusion criteria were: female and male, age over 18, and positive answer to the question ‘Do you use white sugar?’ (Yes, regularly/Sometimes). Other exclusion criteria were: chronic diseases affecting dietary habits or dieting due to obesity, diabetes, celiac disease, or other diseases, as well as medicines taken because of these diseases. Young people were recruited at universities, while other people during public lectures, various events, and at Universities of the Third Age.

### 2.2. Questionnaire

The questionnaire was presented in Table 1. First part of the questionnaire consisted of 19 questions relating to the use of sugar and sweeteners in respondents’ diets. The questions concerned their preferences, decisive factors and the frequency of sugar consumption, the use of sweeteners and products with high sugar content (fruit preserves), and barriers affected by the limitation of sugar consumption, as well as using sweeteners in the diet. Using sweeteners for preparing fruit preserves was taken into account, as preparing fruit preserves at home is a common practice in Poland. Three of the questions were open-ended and the others were closed-ended. The second part of the questionnaire included 5 questions and it concerned respondents’ sociodemographic details (age, sex, education, residence, and their own assessment of their financial situation). 

### 2.3. Data Analysis

The ANOVA test and multi-dimensional cluster analysis was used. Significance of differences between the values was determined at a significance level of *p* < 0.05. The ANOVA test was used to evaluation the effect of sociodemographic factors on habits in the consumption of sugar and sweeteners, as well as products containing them. Multi-dimensional cluster analysis was used to segmentation consumers according to criteria for the selection of sweetened preserves, consumption of products sweetened by stevia and respondents’ opinion about stevia. Ward’s method (distance Euclidean) was used in cluster analysis.

The statistical analysis of the results was performed using Statistica software version 13.1 PL (StatSoft, Krakow, Poland). 

## 3. Results

### 3.1. Characteristics of Respondents

Table 2 presents the characteristic of the persons surveyed. Respondents taking part in the study were divided into similar age and sex groups. According to the criterion of education, the group of respondents with vocational education was the least represented, while the groups of respondents with high secondary and university education included a similar number of members. The majority of respondents lived in big cities. They declared good or average income (79.45%)—Table 2.

### 3.2. The Use of Sugar in Respondents’ Diet and Reasons for Its Limiting

The majority of respondents declared to use sugar in their diet regularly (52.85%) or occasionally (34.5%). A small percentage (12.65%) of those surveyed did not consume sugar at all. Use of sugar in the diet depended on the sex (*p* = 0.000001), age (*p* = 0.000001), education (*p* = 0.000001), and residence (*p* = 0.0000001) as well as financial situation of respondents (*p* = 0.00297). Men, respondents aged 18–40, with vocational education, from small towns, and who declared an average financial situation regularly used more sugar in their diets. While women, persons aged over 60, with university education would significantly more often declare occasional consumption of sugar. 

Respondents would most frequently use sugar for preparing homemade cakes (59.3%), in coffee beverages (40.15%) or tea (29.25%), as well as for preparing fruit preserves (37.25%). Sugar was less frequently used for cooking (16.35%) and other applications in the kitchen (10.95%; other beverages, desserts, cottage cheese spreads etc.). Adding sugar to tea and coffee depended on the respondents’ sex (*p* = 0.00001), age (*p* = 0.0001), and residence (*p* = 0.001) as well as financial situation (*p* = 0.03). Adding sugar to beverages was significantly more often declared by men, respondents with vocational education, living in small cities, and declaring an average financial situation. Men and respondents aged 26–40 would significantly more often declare to use sugar while cooking (*p* = 0.00001). Women would use it mostly for homemade cakes (*p* = 0.00004) and fruit preserves (*p* = 0.00196) and it referred mostly to persons aged over 41 (*p* = 0.000001).

Nearly half of the respondents (46.55%, *n* = 2000) declared they did not use sugar in beverages at all, while 45.7% of respondents would use 1 or 2 spoons of sugar. A limited percentage of respondents used more sugar in their beverages, i.e. 3 spoons (5.9%) or more (1.85%).

The quantity of sugar added to beverages depended on the sex (0.000001), age (*p* = 0.000001), education (*p* = 0.000001), and residence (*p* = 0.000001), as well as the financial situation of respondents (*p* = 0.000001). Adding sugar to beverages was in a way significant from the statistical point of view reduced by women, respondents aged > 40, living in big cities and declaring very good level of income. In turn men, persons aged 26–40, with secondary school education, living in smaller towns or villages, with average financial situation significantly more often declared to add over 3 spoons to their beverages. 

The majority of respondents (98.1%, *n* = 2000) declared to limit the sugar used in their diet. This limitation was motivated by health-related issues (55.55%), less frequently with watching their weight (27.2%) or reasons relating to their senses (11.7%). Other enumerated factors (3.65%) were ideology and religion (refraining from pleasure during Lent) as well as habits. 

Reasons for limiting the use of sugar in the diet depended on the sex (*p* = 0.000001), age (*p* = 0.00001), education (*p* = 0.000001), and financial condition of respondents (*p* = 0.00208). Limiting sugar consumption due to health-related reasons was, in a way significant from the statistical point of view, reported by women, respondents aged over 60, with a university education, and declaring a very good financial situation. Watching body weight was significantly more often quoted by respondents aged 18–25, with university education, and with a very good financial situation. Reasons relating to their senses were of significant importance for men, respondents aged 41-60, with secondary school education, and average financial situation. 

### 3.3. The Use of Sweetened Fruit Preserves in the Diet of Respondents

The majority of respondents (74.25%) would pay attention to the content of sugar in sweetened preserves. They were mostly choosing fruit preserves with low sugar content—79.65%. Of those surveyed, 14.45% would consume fruit preserves with high sugar content. About 6% of respondents did not provide their answer to this question (results not presented in the tables). 

The choice of fruit preserves with low and high sugar content was determined by the sex (*p* = 0.000001), age (*p* = 0.000001), and place of residence (*p* = 0.00671). Fruit preserves with low sugar content were more frequently chosen by women and respondents aged over 41 living in big cities. Fruit preserves with high sugar content were preferred by men and respondents aged 18–40 living in smaller towns and villages. 

Table 3 presents the consumption of sweetened fruit preserves. Fruit jam was the most frequently chosen by respondents. Significantly more often, i.e. every day or once a week, it was used by men and persons aged 18–25 (*p* = 0.000001). Women and persons aged 41–60 would consume jam less often, once or twice a month (*p* = 0.000001). Plum spread was consumed less often, i.e., 2 or 3 times a week, mainly by men and respondents aged over 41 (*p* = 0.00001). The consumption of marmalade was seldom declared, mainly by men and persons aged over 41, who would consume it 2-3 times a week (*p* = 0.00402). Candied fruit was a less popular product, consumed mainly by men aged below 40 who were not well educated. Fruit confiture, syrup, and mousse were the least popular among Polish consumers, used by a very small group of them only once or twice a month (Table 3). Confiture was significantly more often (*p* = 0.0000001) consumed by women and young respondents (aged 18–25). Syrup was significantly more often consumed by the respondents aged 18–25 and 41–60 (*p* = 0.000001). Fruit mousses constituted significantly more often the choice of women (*p* = 0.00094) and persons aged 18–25 (*p* = 0.00013).

**Figure 1:**
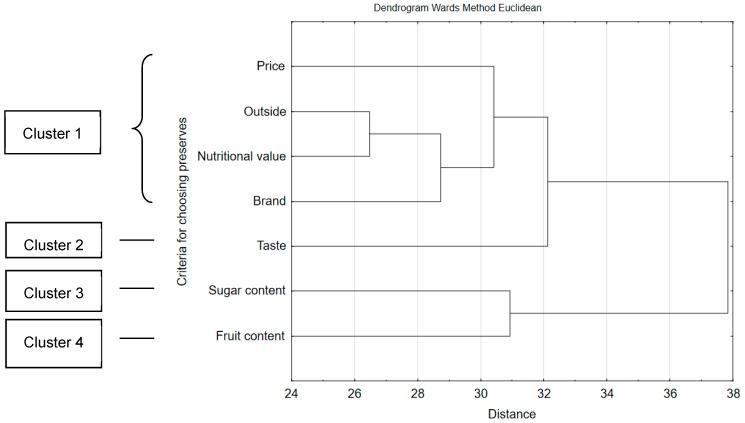
Criteria for the selection of sweetened preserves by the respondents.

While choosing fruit preserves, the consumers would follow different factors. Calculations based on multi-dimensional cluster analysis demonstrate that they can be grouped according to the frequency of indications (Figure 1). Fruit content had the biggest influence on making the choice (cluster 4, 61.75% of consumers), next the taste (cluster 2, 48.5%) as well as sugar content (cluster 3, 42.8%). The following would matter the least for Polish consumers: price, outside, nutritional value and brand (cluster 1, 26.6%).

The criteria for selecting sweetened fruit preserves depended on sociodemographic factors: gender, age, education and financial situation. Consumers’ age was the most decisive for the choice of preserves (*p* < 0.001). Persons aged 18–25 would pay greater attention to the price, taste and outside, while the persons aged 26–40 as well as above 60 to nutritional value, fruit content and brand. The sex was moderately influencing selection criteria (*p* < 0.01). Decisions concerning nutritional value and the outside of the product constituted the exception here, as the sex was insignificant. The study shows that while choosing sweetened preserves, women would significantly more frequently pay attention to fruit and sugar content, while men to the taste, price, and brand (*p* < 0.001). The price was significantly (*p* < 0.001) more often important for the persons residing in small towns, with secondary school education and average financial situation. For the respondents with university education, very good financial situation, and living in big cities, fruit content in the product was more significant (*p* < 0.001). 

In Poland, it is a common tradition to prepare homemade fruit and vegetable preserves, including sweetened fruit preserves. Preparing homemade fruit preserves with low sugar content frequently or occassionaly was declared by as many as 52.5% of respondents, while those with high sugar content by 42.25% of respondents. About 8% of respondents did not provide their answer to this question. 

The type of fruit preserves prepared at home depended on the sex (*p* = 0.00001), age (*p* = 0.00001), education (*p* = 0.001), and place of residence (*p* = 0.001) of the respondents. Jams with low sugar content were significantly more often prepared by women, persons aged over 40, with university education, and living in big cities. Jams with high sugar content were significantly more often prepared by men, persons aged up to 40 (*p* = 0.00001), with secondary school and vocational education, and residing in small towns and villages. 

### 3.4. The Use of Sweeteners and the Level of Knowledge and Use of Products Sweetened with Steviol Glycosides by Respondents

Sweeteners were used by 39.8% of respondents, regularly by 18.05%, and occasionally by 21.75% of those surveyed. Sweeteners were significantly more often selected by persons aged 26–40 (*p* = 0.000001), with university education (*p* = 0.00001), living in big cities (*p* = 0.000001), and declaring good financial situation (*p* = 0.00024).

Sweeteners most frequently used by the respondents were *Stevia Rebaudiana* and xylitol (respectively 26.55% and 23.60%), then aspartame and fructose (15.55% and 12.30% respectively). Other substances, such as acesulfame K, thaumatin and honey were used by a lower percentage of respondents (4.8%) taking part in the survey. 

**Figure 2:**
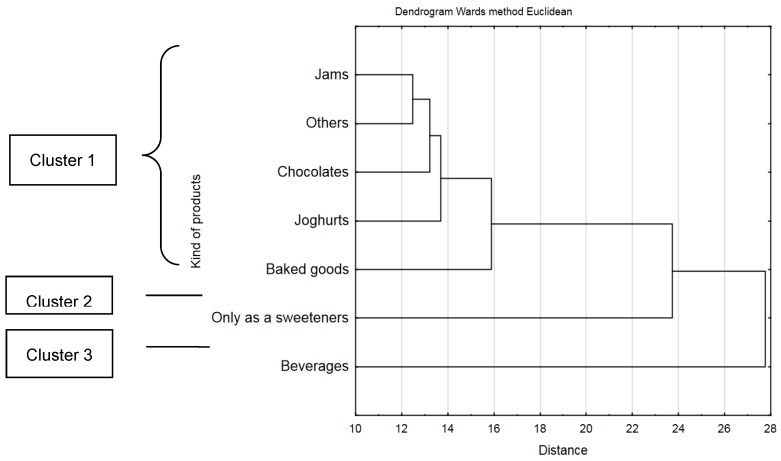
Consumption of products sweetened by stevia among respondents.

The study shows that less than a half of respondents (41.8%, *n* = 2000) consumed products sweetened with stevia. These were: jams, yoghurts, chocolates, beverages, pastries, sweeteners, and other. These products were grouped basing on multi-dimensional cluster analysis depending on the frequency of consumption (Figure 2). Consumers would most frequently use beverages sweetened with stevia (cluster 3, 59.21%), and then use stevia as sweetener (cluster 2, 38.28%). Other products sweetened with stevia, such as jams, chocolates, yoghurts, pastries etc., were the least frequently consumed (cluster 1, 13.08%).

Products sweetened with stevia were significantly more often consumed by the respondents aged 18–40 (*p* = 0.000001), from big cities (*p* = 0.00079), declaring very good financial situation (*p* = 0.00601). Jams and yoghurts sweetened with stevia were significantly more often consumed by respondents aged 18–25 (*p* = 0.001), while chocolate by the respondents with very good financial situation (*p* = 0.00004). Beverages sweetened with stevia were significantly more frequently consumed by men (*p* = 0.00016) and persons aged 26–40 (*p* = 0.000001). Stevia under the form of sweetener was significantly more frequently used by persons from big cities (*p* = 0.00004), and declaring very good financial situation (*p* = 0.000001).

Other products sweetened with stevia were frequently more often consumed by the persons aged 26–40 (*p* = 0.0065). 

An important number of respondents (69.15%, *n* = 2000) declared the willingness to purchase products with lower sugar content and the addition of stevia. Such declarations were significantly more often made by men (*p* = 0.00033) and persons aged 26–40 (*p* = 0.000001).

**Figure 3:**
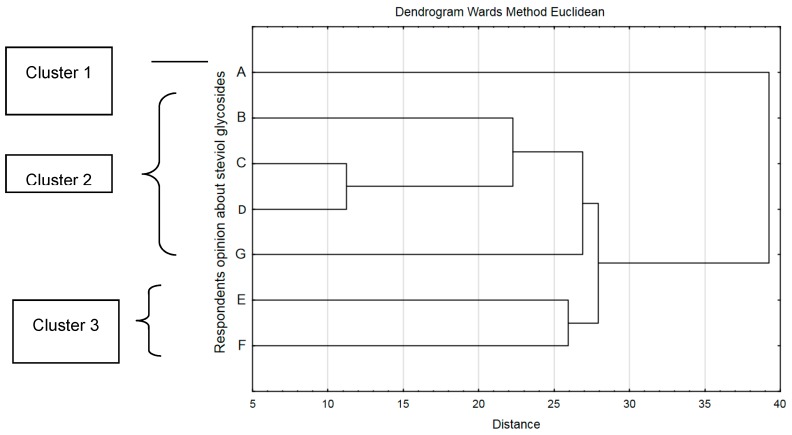
Respondents’ opinion about steviol glycosides. **A**—I have no concerns about the consumption of stevia; **B**—The lack of knowledge on this subject raises my concerns; **C**—It can change the taste of product, in particular the aftertaste of bitterness will appear; **D**—I avoid highly processed products; **E**—May cause allergy and diarrhea; **F**—I have no opinion on this subject; **G**—I like natural and traditional products.

Stevia as a sweetener has become popular in products available on the market as late as in 2011. For this reason, the opinion of Polish consumers concerning this sweetener has been studied (Figure 3). Based on the calculations made with the use of multi-dimensional cluster analysis it was stated that their opinions varied. Three consumer groups having distinct opinions about the presence of stevia in products available on the market were differentiated. The first group, the biggest of them, did not present any concerns in connection with using stevia (cluster 1, 38.95%). The second group of consumers expressed very diversified opinion in this field (cluster 2, 24.2%). The third group, the smallest one, was afraid of the negative impact that stevia may have on people’s health (cluster 3, 13.35), for example diarrhea or allergy. Consumers from group 2 did not have any opinion or knowledge about stevia, they would state that they prefer natural products or were afraid that the addition of stevia may influence the product’s taste or cause bitter aftertaste. 

Among the persons expressing concerns connected with consuming products containing stevia, those aged 60 and over (*p* = 0.00014) prevailed. The lack of knowledge concerning stevia was significantly more often presented by men (*p* = 0.00222) and respondents aged above 40 (*p* = 0.000001). Aftertaste (bitterness) was significantly more frequently the concern of respondents aged 18–25 (*p* = 0.000001), with secondary school education (*p* = 0.00063). Persons with the lowest level of education were significantly more often afraid of allergy (*p* = 0.00722). Respondents aged over 40 significantly more often did not have any opinion about stevia or did not know that it is a natural substance (0.000001). What is more, women would significantly more frequently emphasize that they avoid the consumption of processed food (*p* = 0.02298).

Majority of respondents (98.1%, *n* = 2000) declared to try to limit use of sugar in their diet. The reasons (barriers) of limitation of the sugar in respondents’ diet were the lack of information about food safety of sweeteners (31%), the lack of money for buying an innovation food (12%), choosing rather natural products (4%), as well as habits of eating sugar (78%) by respondents. 

## 4. Discussion

The conducted studies made it possible to evaluate the consumption of sugar and sweeteners in the diet of Polish consumers. The majority of respondents (87.4%) used sugar in their diet with various frequencies. At present, excessive consumption of added sugar together with different products motivates food experts to limit it in the diet, which is expressed in dietary guidelines. WHO recommends to limit the consumption of sugar to less than 10% of daily energy demand, encouraging to reduce the consumption to the level below 5% [4].

Polish men and young consumers aged 18–25 use the biggest quantities of sugar in their diet. They would most often act contrary to the guidelines, e.g., consume products with high sugar content, both those available on the market as well as prepared in their households. They consumed jams every day or once a week. What is more, they would add 3 or more teaspoons of sugar to their coffee and tea and use sugar while cooking. The similar situation accompanying increased consumption of sweetened coffee/tee among Canadian adolescents was reported by Godin et al. [28]. In the group of Polish respondents, sugar intake exceeded the level recommended by WHO [4], i.e. no more than 25 g (6 teaspoons) daily. The similar results were obtained by Mesana et al. [29] in European adolescents. Total sugars intake represented 23.6% and free sugars 19% of energy intake. 

The group of Polish young consumers was characterized by different attitude towards limiting sugar in their diet. If they did limit sugar, it was due to weight watching reasons. Persons aged 18–25 are more open to new things and they would significantly more frequently consume products sweetened with stevia, and men declared a willingness to consume them in the future, even if they significantly more often declared a lack of knowledge about stevia and had some concerns in this field. This group may constitute potential clients for products sweetened with natural sweeteners, such as for example steviol glycosides. Our findings confirmed the hypothesis that consumers are limiting the added sugar in their diet.

It is necessary to emphasize that Polish consumers more and more often pay attention to healthy behavior and limit added sugar in their diet or use saccharose substitutes [30]. This results from the change in their lifestyle. However, their diets are characterized by a higher content of recommended food groups, i.e. sweets and beverages with added sugar [27]. Many authors reported that gender and age played direct roles in predicting nutrition concern [31,32]. Women and older people are more concerned about healthy behavior than men and younger people [33]. Additionally, older people appear to be more concerned about food issues [33]. Similar mechanisms were observed among Polish consumers. But in the research of Czlapka-Matyasiak et al. [34], conducted among young women, it was found the quality of diet was compromised regardless of restricting or not restricting sugar during the weekend.

Polish women and persons aged over 60 used sugar occasionally or would limit it due to health reasons. The most frequent way to limit the amount of sugar in the diet was to resign from adding it to their coffee and tea and to buy fruit preserves with low sugar content. However, they significantly more often used sugar for preparing homemade cakes and preserves. Women and the elderly followed different criteria while purchasing fruit preserves available on the market. Fruit and sugar content were more important for women, while the elderly paid greater attention to their calorific value. Women preparing sweetened preserves in their household also declared the preparation of their variants with low sugar content. However, both women as well as the elderly do not choose such functional novelties as the products sweetened with stevia. Elder respondents would significantly more often express their concerns with regard to the consumption of stevia and did not know this sweetener. Women in turn did not treat this sweetener as natural and would significantly more often declare to avoid highly processed food.

Opposite to our results, in the research of other authors, consumption of low-calorie sweeteners increases with age [7,35,36,37]. It is most common among adults between 55 and 74 years of age compared to younger individuals. Low-calorie sweeteners consumption also associated with socio-economic status and educational attainment, with higher income families reporting the highest prevalence of sweeteners use [35]. Female adolescents 12–19 years of age consumed more sweeteners than their male counterparts [7]. According to Sylvetsky and Rother [37] sucralose has become the most commonly used low-calorie sweetener, while other sweeteners (e.g., aspartame) are becoming less popular. Concurrently, popularity of newer and natural sweeteners, as stevia, is increasing. Consumption of low-calorie sweeteners varies according to age, race/ethnicity, and socio-demographic factors. White, older, and more educated consumers consume more of them. Such correlations were not found in own studies, because in Poland stevia has been known for only a few years and products sweetened with stevia are still rare on the market. 

Apart from the age and sex, other factors influence the reduction of sugar consumption in favor of sweeteners, including products containing stevia. These were education, place of residence and financial situation. Consumers’ declarations on limiting sugar consumption would increase with better education, financial situation and city size. These respondents were significantly more often choosing sweeteners, including stevia and the products with its addition. They would also opt for products with low sugar content, including those available on the market as well as homemade. While purchasing fruit preserves, fruit content in the product was significantly more often taken into consideration. Similar correlations are presented by Cardoso and Bolini [38]. According to these authors, there is a growing trend towards the consumption of diet and light products. These products are indicated, among other purposes, for people with diabetes or other medical restrictions, including obesity and for people who are concerned with aesthetics and health.

It is necessary to emphasize that products containing steviol glycosides still constitute a novelty on the Polish market of functional food and the market itself is not big. The studies demonstrate that consumers’ knowledge in this field is limited and some of them, for example the elderly and men, even express some concerns in connection with their consumption. And as it is known from other studies [39], while selecting and approving functional foods, what matters a lot these are consumers’ lifestyle attitudes in the field of health benefits and risks as well as preventing diseases through consuming the foods containing healthy ingredients. 

Our studies demonstrate that insufficient knowledge of Polish consumers concerning steviol glycosides is the source of low interest of both women as well as persons aged over 60 in stevia and products containing its addition. The following factors presented by the authors [40] may influence their acceptance as innovative products: perceiving the product from the perspective of benefits and risks connected with it, quality and price as well as other characteristics of the product, e.g., its natural character. Consumers present their trust in natural food and suspicious attitude towards new technologies [41]. With increased consumer interest in reducing sugar intake, food products made with sweeteners rather than sugar have become more popular [42]. Additionally, consumption of stevia has demonstrated to be generally safe in most reports [43].

The conducted studies made it possible to present the characteristics of the consumption of sugar and sweeteners in the diet of Poles. Consumer groups to which promotional campaigns concerning the consumption of stevia and nutrition campaigns on the necessity to limit the content of sugar in the diet are worth addressing were identified. Those factors influencing consumers’ preferences and opinions which can be verified and assessed were studied. 

### Strengths and Limitations

The strength of the study is its relatively large sample (*n* = 2000). This paper is one of a few about consumption of sugar and sweeteners by Polish consumers. The following factors were not taken into account: individual (liking of sugary food), interpersonal (attitudes of peers), and environmental factors (media, health professionals, and food labeling). They can influence the adults’ knowledge and attitudes about sugar, but difficult to established an association between knowledge and attitudes about sugar and sugar intake [44]. The fact of this subject not being analyzed may constitute a limitation of presented studies. Despite these limitations, the present paper contributes to the growing body of research concerning the evaluation of the use of sugar and selected sweeteners on the example of Poles diet. 

## 5. Conclusions 

Sugar consumption among surveyed Poles remained at a high level. It was influenced by many different factors, such as age, sex, education, and place of residence. However, positive trends were observed in connection with changing the attitude towards sugar consumption in the diet. Polish consumers declared to be undertaking activities aiming at reducing the amount of sugar in their diet, mainly due to health-related reasons. It was emphasized mainly by women taking part in the survey. 

Fruit preserves with high sugar content do not enjoy big popularity among the surveyed group of consumers. However, the studies made it possible to identify consumer groups who still consume large quantities of these products and this is the group to which adapted information campaigns leading to the change in their lifestyle should be addressed. These were men, persons aged 18–40 and living in smaller towns and villages. 

The most frequent way to limit the quantity of sugar in the diet consisted in the use of sweeteners, mainly stevia and xylitol. Products sweetened with stevia were consumed by less than half of consumers. These were mainly beverages and sweeteners. The respondents are characterized by limited, and even incorrect knowledge about stevia. Apart from the identified group of consumers who did not present any concerns relating to the consumption of stevia, the remaining groups did not know enough about it or did not express their positive opinion. According to them, the addition of stevia may have a negative influence on the product’s taste, or they did not consider stevia to be a natural product. A positive thing consisted in the fact that only a limited group of consumers linked the consumption of stevia with negative influence on health. 

The conducted studies are the source of up-to-date information about the consumption of sugar and sweeteners. They may be useful for dieticians, educational institutions, and representatives of industry for establishing successful informative and health promotion campaigns. Their aim would be to increase consumers’ knowledge and awareness connected with excessive sugar consumption and the benefits of using sweeteners, including stevia.

## Figures and Tables

**Table 1:** Questionnaire.

No	Questions	Variants of Answers
1	Do you use white sugar?	Yes, regularly/No/Sometimes
2	What do you use most often sugar for?	For sweetening tea
		For sweetening coffee
		For preparation of baked goods
		For cooking and culinary use
		For preparing fruit preserves
3	How many teaspoons of sugar do you use most often to sweeten beverages?	I do not sweeten
		I sweeten with 1, 2, 3, 4, or more teaspoon
4	Do you limit sugar in your diet?	Yes/No
5	For what reasons do you limit sugar in your diet?	Health-related issues
		Watching the weight
		Sensory aspects
		Others, what…..
6	What are the reasons for which you do not limit sugar in your diet?	Habits of eating sugar
		Food safety related to the consumption of sweeteners
		Consumption of natural products
		Lack of money for innovation of food
7	Do you replace sugar with sweetener?	Yes/No/Sometimes
8	Which type of sweetener do you use most often?	fructose, xylitol, stevia, aspartame, acesulfame K., thaumatin, others (e.g., honey)
9	Do you eat jam?	Yes/No/Sometimes
10	What criteria do you follow when choosing jam? Please mark out the three most important ones	Price/taste/outside/nutritional value/brand/sugar content/fruit content
11	Which of the mentioned sweetened food is most often consumed in your household? jam marmalade plum spread candied fruits fruit confiture syrup fruit mousses other……….	every day/2 or 3 times a week/once a week/ 5 or 6 times a month/1 or 2 times a month/no consumption
12	Do you pay attention to the sugar content in jam?	Yes/No
13	What jams do you choose most often?	low-sugar jams/high-sugar jams
14	Do you make jam at home by yourself?	Yes/No/Sometimes
15	What kind of jams do you make at home?	low-sugar jams/high-sugar jams
16	Do you willingly buy new products on the sweetened products market?	Yes/No
17	Have you ever consumed products sweetened with stevia?	Yes/No
18	Which of the products sweetened with stevia have you ever consumed?	jam/chocolate/beverages/baked goods/yoghurts/only sweetener/others……….
19	Would you like to buy a product with reduced sugar content with the addition of stevia?	Yes/No
20	Gender	Women/men
21	Age	18–25 years old/26–40 years old/41–60 years old/over 60 years old
22	Education	vocational and elementary school
		secondary school/higher education (university)
23	Residence	city over 500 000 inhabitants
		city up to 100 000 inhabitants and village
24	Financial status	very good/good/average

**Table 2:** Characteristic of the evaluated population.

Features of Population	Group	Number of Respondents (*n*)	Percentage of Respondents (%)
Total	-	2000	100
Gender	women	1089	54.45
	men	911	45.55
Age	18–25 years old	500	25.00
	26–40 years old	500	25.00
	41–60 years old	500	25.00
	over 60 years old	500	25.00
Education	vocational and elementary school	308	15.40
	secondary school	867	43.35
	higher education (university)	825	41.25
Residence	city over 500 000 inhabitants	1245	62.25
	city up to 100 000 inhabitants and village	755	37.75
Financial situation	very good	411	20.55
	good	899	44.95
	average	690	34.50

**Table 3:** Frequency of consumption of sweetened fruit preserves by the respondents.

Kind of Preserves	Percentage of Answers (%, *n* = 2000)						Average ± SD(MMedian)
	Every Day	2 or 3 times a Week	Once a Week	5 or 6 times a Month	1 or 2 times a Month	No Consumption	
Jams	2.60	10.20	14.95	24.80	34.95	12.50	4.17 ± 1.27 (4)
Marmalade	0.05	1.50	2.90	8.65	33.25	53.65	5.35 ± 0.87 (6)
Plum spread	0.70	4.80	8.40	18.20	40.55	27.35	4.75 ± 1.13 (5)
Candied fruit	0.65	1.80	2.90	8.20	35.05	51.40	5.29 ± 0.94 (6)
Fruit confiture	0.15	2.40	5.80	17.20	40.40	34.05	4.97 ± 0.99 (5)
Syrup	0.95	5.20	6.85	12.50	31.35	43.15	4.98 ± 1.20 (5)
Fruit mousses	0.70	3.30	6.75	15.25	30.65	43.35	5.02 ± 1.12 (5)

Scale: 1—every day, 2—2 or 3 times a week, 3—once a week, 4—5 or 6 times a month, 5—once or twice times a month, and 6—no consumption.

## References

[1] (1972–2017). Statistical Yearbook of the Republic of Poland 1971–2016.

[2] Institute of Agricultural and Food Economics, Agricultural Market Agency & Ministry of Agriculture and Rural Development (2016). Sugar Market: Currant state and perspective. Anal. Rynkowe.

[3] OECD-FAO Agricultural Outlook OECD Agriculture Statistics *(Database)*.

[4] World Health Organization (2015). Guideline: Sugars Intake for Adults and Children.

[5] World Health Organization (2015). WHO Calls on Countries to Reduce Sugars Intake among Adults and Children.

[6] Popkin B.M., Hawkes C. (2016). The sweetening of the global diet, particularly beverages: Patterns, trends and policy responses for diabetes prevention. Lancet Diabetes Endocrinol..

[7] Fakhouri T.H., Kit B.K., Ogden C.L. (2012). Consumption of diet drinks in the United States, 2009–2010. NCHS Data Brief..

[8] Imamura F., O’Connor L., Ye Z., Mursu J., Hayashino Y., Bhupathiraju S.N., Forouhi N.G. (2015). Consumption of sugar sweetened beverages, artificially sweetened beverages, and fruit juice and incidence of type 2 diabetes: Systematic review, meta-analysis, and estimation of population attributable fraction. BMJ.

[9] Te Morenga L., Mallard S., Mann J. (2013). Dietary sugars and body weight: Systematic review and meta-analyses of randomised controlled trials and cohort studies. BMJ.

[10] Centers for Disease Control and Prevention (CDCP) (2012). Chronic Diseases and Health Promotion.

[11] Stanhope K.L. (2016). Sugar consumption, metabolic disease and obesity: The state of the controversy. Crit. Rev. Clin. Lab. Sci..

[12] Malik V.S., Hu F.B. (2015). Fructose and cardiometabolic health. J. Am. Coll. Cardiol..

[13] Augustin L.S.A., Kendall C.W.C., Jenkins D.J.A., Willett W.C., Astrup A., Barclay A.W., Björck I., Brand-Miller J.C., Brighenti F., Buyken A.E. (2015). Glycemic index, glycemic load and glycemic response: An International Scientific Consensus Summit from the International Carbohydrate Quality Consortium (ICQC). Nutr. Metab. Cardiovasc. Dis..

[14] Bray G.A., Popkin B.M. (2014). Dietary sugar and body weight: Have we reached a crisis in the epidemic of obesity and diabetes?: Health be damned! Pour on the sugar. Diabetes Care.

[15] O’Connor L., Imamura F., Brage S., Griffin S.J., Wareham N.J., Forouhi N.G. (2018). Intakes and sources of dietary sugars and their association with metabolic and inflammatory markers. Clin. Nutr..

[16] Pawar R.S., Krynitsky A.J., Rader J.I. (2013). Sweeteners from plants with emphasis on *Stevia rebaudiana* (Bertoni) and *Siraitia grosvenorii* (Swingle). Anal. Bioanal. Chem..

[17] Yadav A.K., Singh S., Dhyani D., Ahuja P.S. (2011). A review on the improvement of stevia [*Stevia rebaudiana* (Bertoni)]. Can. J. Plant Sci..

[18] Mooradian A.D., Smith M., Tokuda M. (2017). The role of artificial and natural sweeteners in reducing the consumption of table sugar: A narrative review. Clin Nutr ESPEN.

[19] Al-Qudsi F.M., Al-Hasan M.M. (2019). In utero exposure to commercial artificial sweeteners affects mice development and mammary gland structure. Environ. Sci. Pollut. Res. Int..

[20] Kim J.Y., Seo J., Cho K.H. (2011). Aspartame-fed zebrafish exhibit acute deaths with swimming defects and saccharin-fed zebrafish have elevation of cholesteryl ester transfer protein activity in hypercholesterolemia. Food Chem. Toxicol..

[21] Kinghorn A.D., Chin Y.W., Pan L., Jia Z., Mander L., Liu H.-W. (2010). Natural Products as Sweeteners and Sweetness Modifiers. Comprehensive Natural Products II: Chemistry and Biology.

[22] (2011). Commission Regulation (EU) No 1131/2011 of 11 November 2011, amending Annex II to Regulation (EC) No 1333/2008 of the European Parliament and of the Council with regard to steviol glycosides. Off. J. Eur. Union.

[23] European Food Safety Authority (EFSA) (2010). Scientific opinion on the safety of steviol glycosides for the proposed uses as a food additive. EFSA J..

[24] United States Department of Agriculture (USDA) (2012). Sugar and Sweeteners Outlook: June 2012. USDA No. (SSSM-286). https://www.ers.usda.gov/publications/pub-details/?pubid=39310.

[25] Christaki E., Bonos E., Giannenas I., Karatzia M.A., Florou-Paneri P.F. (2013). *Stevia rebaudiana* as a novel source of food additives. J. Food Nutr. Res..

[26] González C., Tapia M., Pérez E., Pallet D., Dornier M. (2013). Main properties of steviol glycosides and their potential in the food industry: A review. Fruits.

[27] Basiak A., Różańska D., Połtyn-Zaradna K., Wołyniec M., Szuba A., Zatońska K. (2018). Comparison of intake of food groups between participants with normoglycemia, impaired fasting glucose, and type 2 diabetes in PURE Poland population. Int. J. Diabetes Dev. Ctries..

[28] Godin K.M., Hammond D., Chaurasia A., Leatherdale S.T. (2018). Examining changes in school vending machine beverage availability and sugar-sweetened beverage intake among Canadian adolescents participating in the COMPASS study: A longitudinal assessment of provincial school nutrition policy compliance and effectiveness. Int. J. Behav. Nut. Phys. Act..

[29] Mesana M.I., Hilbig A., Androutsos O., Cuenca-Garcia M., Dallongeville J., Huybrecht I., De Henauw S., Widhalm K., Kafatos A., Nova E. (2018). Dietary sources of sugars in adolescents’ diet: The HELENA study. Eur. J. Nutr..

[30] Sajdakowska M., Jankowski P., Gutkowska K., Guzek D., Żakowska-Biemans S., Ozimek I. (2018). Consumer acceptance of innovations in food: A survey among Polish consumers. J. Consum. Behav..

[31] Burton M., Chun Wang W., Worsley A. (2015). Demographic and psychographic associations of consumer intentions to purchase healthier food products. Prev. Med. Rep..

[32] Rollin F., Kennedy J., Wills J. (2011). Consumers and new food technologies. Trends Food Sci. Technol..

[33] Worsley A., Scott V. (2000). Consumers’ concerns about food and health in Australia and New Zealand. Asia Pac. J. Clinic. Nutr..

[34] Czlapka-Matyasik M., Lonnie M., Wadolowska L., Frelich A. (2018). “Cutting Down on Sugar” by Non-Dieting Young Women: An Impact on Diet Quality on Weekdays and the Weekend. Nutrients.

[35] Drewnowski A., Rehm C.D. (2015). Socio-demographic correlates and trends in low-calorie sweetener use among adults in the United States from 1999 to 2008. Eur. J. Clin. Nutr..

[36] Piernas C., Ng S.W., Popkin B. (2013). Trends in purchases and intake of foods and beverages containing caloric and low-calorie sweeteners over the last decade in the United States. Pediatr. Obes..

[37] Sylvetsky A.C., Rother K.I. (2016). Trends in the consumption of low-calorie sweeteners. Physiol. Behav..

[38] Cardoso J.M.P., Bolini H.M.A. (2007). Different sweeteners in peach nectar: Ideal and equivalent sweetness. Food Res. Int..

[39] Jeżewska-Zychowicz M. (2009). Impact of beliefs and attitudes on young consumers’ willingness to use functional food. Pol. J. Food Nutr. Sci..

[40] De Steur H., Gellynck X., Storozhenko S., Liqun G., Lambert W., Van Der Straeten D., Viaene J. (2010). Willingness-to-accept and purchase genetically rice with folate content in Shanxi Province, China. Appetite.

[41] Huotilainen A., Tuorila H. (2005). Social representation of new foods has a stable structure based on suspicion and trust. Food Qual. Prefer..

[42] Pinheiro M.V.S., Oliveira M.N., Penna A.L.B., Tamime A.Y. (2005). The effect of different sweeteners in low-calorie yogurts-a review. Int. J. Dairy Technol..

[43] Rojas E., Bermúdez V., Motlaghzadeh Y., Mathew J., Fidilio E., Faria J., Rojas J., de Bravo M.C., Contreras J., Mantilla L.P. (2018). *Stevia rebaudiana Bertoni* and Its Effects in Human Disease: Emphasizing Its Role in Inflammation, Atherosclerosis and Metabolic Syndrome. Curr. Nutr. Rep..

[44] Gupta A., Smithers L.G., Harford J., Merlin T., Braunack-Mayer A. (2018). Determinants of knowledge and attitudes about sugar and the association of knowledge and attitudes with sugar intake among adults: A systematic review. Appetite.

